# Comparison of clinical outcomes between pulsatile and non-pulsatile perfusion during cardiopulmonary bypass: A prospective randomized study

**DOI:** 10.1051/ject/2026007

**Published:** 2026-06-19

**Authors:** Aakriti Sharma, Prajjwol Luitel, Prabhat Khakural, Ranjan Sapkota

**Affiliations:** 1 Department of Cardiothoracic and Vascular Surgery, Manmohan Cardiothoracic Vascular and Transplant Center, Maharajgunj Kathmandu Nepal; 2 Maharajgunj Medical Campus, Institute of Medicine Kathmandu Nepal

**Keywords:** Atrial septal defect, Cardiopulmonary bypass, Cardiac surgery, Microcirculation, Pulsatile perfusion, Perfusion physiology

## Abstract

*Background*: The clinical benefits of pulsatile perfusion during cardiopulmonary bypass (CPB) remain a subject of debate. This study aimed to compare clinical outcomes between pulsatile and non-pulsatile perfusion during CPB in patients undergoing elective cardiac surgery. *Materials and methods*: Eighty patients undergoing elective atrial septal defect (ASD) closure or mitral valve replacement (MVR) between July 2019 and July 2020 were randomized to two groups: pulsatile perfusion (PP) group and non-pulsatile perfusion (NP) group. All surgeries were performed by a single surgical team. In the PP group, pulsatile flow was maintained at 60–80 per minute for adults and 80–120 per minute for pediatric patients, pulse pressure was maintained higher than 15 mm Hg, while all other intraoperative management and perioperative protocols were kept consistent between groups. The two groups were compared in terms of length of ICU stay, hospital stay, renal function test, liver function test, and complications. *Results*: 77 patients with a mean age of 33.8 ± 15.9 were included in the final analysis. The groups were similar in demographics, clinical and intra-operative variables, except body surface area and baseline creatinine, which were higher in the pulsatile group. Length of ICU stay, hospital stay, liver function tests, renal function tests, and complications were similar across both groups, except for higher urea (*p*-value 0.049) in the pulsatile group. *Conclusions*: This study did not demonstrate significant differences in clinical outcomes between pulsatile and non-pulsatile flow during CPB in elective cardiac surgery. Routine use of pulsatile flow cannot be recommended based on current findings. The study was limited by its restriction to patients undergoing ASD closure or MVR surgery and the dominance of younger patients without major comorbidities.

## Introduction

The modern era of cardiac surgery commenced in the early 1950s with the introduction of the cardiopulmonary bypass (CPB) technique [1, 2]. Significant efforts have been dedicated to developing strategies to minimize the inflammatory response associated with CPB [1, 2]. Earlier, non-pulsatile perfusion was a routine perfusion technique during CPB due to its simplicity and technical feasibility [3, 4]. Currently, pulsatile perfusion is gaining popularity due to its ability to mimic the pulsatile nature of blood flow, resulting in improved microcirculation, decreased systemic vascular resistance, improved oxygen delivery, and consumption [5].

There has been an ongoing controversy over the benefits of pulsatile and non-pulsatile perfusion during CPB for over half a century, with some studies [6, 7] favoring pulsatile perfusion and some [8–10] reporting no significant differences between the perfusion techniques. Non-pulsatile perfusion remains a routine clinical practice in most institutions, including ours, due to its simplicity and technical feasibility. Although the physiologic rationale for pulsatile perfusion is compelling, the degree to which its benefits translate into clinical differences depends on patient characteristics and procedures. In younger patients with few comorbidities and relatively short CPB and cross-clamp durations, the clinically meaningful differences may be limited [11]. In low- and middle-income settings such as ours, cardiac surgery is dominated by valve surgery rather than coronary bypass procedures, resulting in a case mix that differs from that of many high-income centers. This prospective randomized controlled study aimed to compare clinical outcomes of pulsatile and non-pulsatile perfusion in patients undergoing elective cardiac surgeries in a tertiary care center of Nepal.

## Materials and methods

### Study design

**Figure 1:**
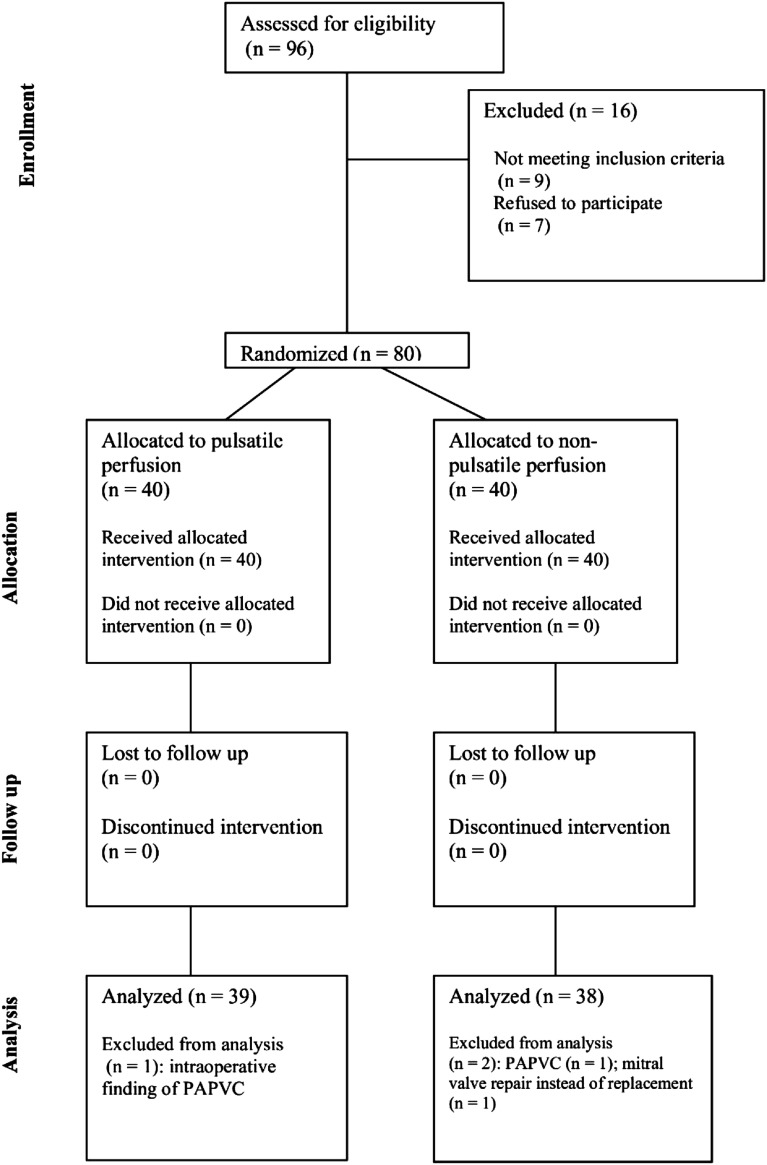
CONSORT flow diagram.

This was a prospective, randomized study conducted following the ethical guidelines of the Declaration of Helsinki 2013 and has been reported in line with the CONSORT 2025 criteria (Figure 1) [12].

### Setting and population

The study was conducted at the Department of Cardiothoracic and Vascular Surgery, Manmohan Cardiothoracic Vascular and Transplant Center, Maharajgunj, Kathmandu, from July 2019 to July 2020. It performs 350–400 on-pump cases annually and is one of two tertiary care cardiac centers in Nepal. Consecutive patients undergoing open heart surgery for repair of Atrial Septal Defect (ASD) closure and Mitral Valve replacement (MVR) were included.

### Sample size

The sample size was calculated using the formula: *N* = 4*pq*/*l*^2^, where *p* is the prevalence, *q* = 1 − *p*, and *l* is the allowable margin of error. The prevalence of 15.6% for ASD with MVR procedures was calculated from the audit record of the previous year at our institution. With an allowable error of 5% and a confidence level exceeding 95%, the required sample size was 77 patients. The required sample size was divided into two groups, with 38 participants in one arm and 39 in the other.

### Inclusion and exclusion criteria

All consecutive patients scheduled for elective ASD closure or MVR with normal left ventricular ejection fraction (LVEF) who provided written informed consent were eligible for inclusion. Patients with moderate to severely reduced LVEF, bleeding disorders, acute kidney injury (AKI), impaired liver function, or those who declined to provide written informed consent were excluded. In addition, procedures other than isolated ASD closure or MVR (e.g., partial anomalous pulmonary venous connection [PAPVC] repair or mitral valve repair) were excluded to maintain a homogeneous study population with comparable operative characteristics.

### Methodology

Before surgery, all patients underwent a standardized preoperative evaluation, including detailed history-taking, physical examination, and relevant investigations. Randomization was performed using a computer-generated sequence created by an independent statistician who was not involved in patient recruitment or outcome assessment. Allocation codes were placed in sequentially numbered, opaque, sealed envelopes. After confirming eligibility and obtaining written informed consent, the operating room nurse opened the next envelope immediately before surgery to assign the patient to either the pulsatile perfusion (PP) or non-pulsatile perfusion (NP) group, ensuring allocation concealment up to the time of operation. Surgeons, anesthetists, and perfusionists were aware of group assignment; however, postoperative clinical data were collected using a predefined proforma, and laboratory investigations were processed in the central hospital laboratory according to routine protocols. Outcome assessors and data analysts were not formally blinded, but the primary endpoints (ICU and hospital length of stay, renal and liver function tests, and major complications) were objective.

Every patient underwent the same surgical procedure, perfusion protocol, and postoperative management. Induction was done using intravenous midazolam (0.1–0.2 mg/kg) and ketamine (2 mg/kg), and muscle relaxation was done with pancuronium (0.1–0.2 mg/kg). Analgesia was provided by fentanyl (50 μg/kg) before initiating CPB. Patients received either pure oxygen or a blend of oxygen and air for ventilation, with isoflurane added if tolerated. Monitoring included noninvasive techniques such as ECG, pulse oximetry, and respiratory gas measurements, along with invasive monitoring of arterial and central venous pressures. Phentolamine (0.1 mg/kg) was administered to manage peripheral vascular resistance during cooling and rewarming on bypass. Discontinuation of CPB involved bolus doses of calcium gluconate and dopamine infusions. Postoperative evaluations were done daily through clinical assessments and laboratory tests. Extubation criteria included age-appropriate mean arterial pressure, FIO_2_ ≤ 40%, stable arterial blood gases, and a urine output of at least 2 mL/kg/h.

### CPB technique

All patients received systemic heparinization before cannulation, with unfractionated heparin administered at 300–400 units/kg. CPB was performed using a standard roller-pump system (Jostra HL20 or Sorin S5 heart-lung machine) with a non-coated, custom-made perfusion pack and a hollow-fiber membrane oxygenator. The extracorporeal circuit also included a myocardial protection unit for administration of Del Nido cardioplegia. Following median sternotomy, arterial cannulation was performed via the ascending aorta using an aortic cannula (22–24 Fr) selected according to the patient’s body surface area and flow requirements. Bicaval venous cannulation was achieved using either straight or angled cannulas. Throughout the bypass period, normothermia was maintained with a target systemic temperature of 34–36 °C. A cardiac index of 2.2–2.4 L/min/m^2^ and mean arterial pressure (MAP) of 60–80 mm Hg were targeted.

In the NP group, the roller pump operated in continuous flow mode. In the PP group, perfusion began in non-pulsatile mode and was switched to pulsatile mode once native ventricular ejection ceased. Pulsatile flow was generated using the roller pump head fitted with tubing of 5/8 inch × 3/32-inch diameter. A base flow of 2.2–2.4 L/min/m^2^ was maintained, with a pulse rate of 60–80 per minute for adults and 80–120 per minute for pediatric patients. The active pump time constituted 50–55% of each cycle, maintaining a pulse pressure greater than 15 mm Hg. Although this pulse pressure was relatively modest, the MAP of 60–80 mm Hg ensured adequate renal and cerebral perfusion under normothermic conditions while preventing excessive pressure fluctuations within the circuit. In the PP group, pulsatile flow was initiated immediately after native cardiac ejection ceased and continued until spontaneous ejection resumed during weaning from bypass. The duration of pulsatile perfusion, therefore, varied among cases depending on the total CPB time, corresponding approximately to 50–55% of the total bypass cycle in most patients.

At the end of CPB, vasopressors and inotropes were administered as clinically indicated. Postoperative management followed a standardized protocol in the dedicated cardiac surgical recovery unit and inpatient ward.

### Data collection

Data was collected and entered in a pre-designed proforma by surgery residents. Variables that could affect outcomes were recorded: Preoperative factors were age, gender, body mass index (BMI), total body surface area (BSA), previous cardiothoracic surgery, renal function, and liver function tests. The intraoperative factors were CPB time, aortic cross-clamp time, urine output, and arterial blood gas analysis (ABG) findings. Post-operatively: dose, duration of vasopressors, inotropes, renal function (RFT), liver function test (LFT), urine output, and drain output were recorded. Outcome variables included length of intensive care unit (ICU) stay, post-surgery hospital stay, complications, and outcomes were recorded.

### Outcomes

The primary objective was to compare clinical outcomes, namely ICU stay, hospital stay, LFT, RFT, and postoperative complications between the PP and NP groups. The secondary objectives were to compare the intraoperative variables (CPB time, aortic cross-clamp time, urine output, arterial blood gases, and lactate) between pulsatile and non-pulsatile CPB groups, and to assess the requirement and duration of vasopressors and inotropes postoperatively in both groups.

### Statistical analysis

Statistical analysis was conducted using the IBM Statistical Package for the Social Sciences (version 24; IBM Corporation, Chicago, IL, USA). Continuous variables were presented as mean ± standard deviation (SD) or median [Inter-quartile range (IQR)] for normally and non-normally distributed variables. The Shapiro–Wilk test was run to see whether it was normally distributed (*p*-value > 0.05) or not (*p*-value < 0.05). Two groups were compared using an independent samples *t*-test or Mann–Whitney U test for normal and non-normally distributed data. Categorical variables were compared using the Chi-square test or the Fisher’s exact test. A *p*-value of ≤ 0.05 was considered statistically significant.

### Ethical consideration

The study protocol was approved by the Institutional Review Committee of the Institute of Medicine (20.7.2017). Before enrolment, patients provided informed consent. Anonymity and confidentiality of data were maintained.

## Results

Of the 40 patients initially enrolled in each group, one patient in the pulsatile group was excluded due to an intraoperative finding of PAPVC, and two patients in the non-pulsatile group were excluded because one had PAPVC and one underwent mitral valve repair instead of replacement (Figure 1).

### Demographic and clinical characteristics

The groups were similar in demographics and clinical characteristics, except for BSA and baseline creatinine, which were higher in the PP group (Table 1).

**Table 1:** Demographic and clinical characteristics of pulsatile and non-pulsatile CPB groups.

	Pulsatile group (*n* = 39)	Non-pulsatile group (*n* = 38)	*p*-Value
*Demographics*			
Age in years (Mean ± SD)	35.5 ± 15.8	32 ± 16.1	0.34
Males (%)	12 (30.8%)	13 (34.2%)	0.81
Weight (in kg)	50.4 ± 17.7	47.3 ± 16.4	0.43
Height (in cm)	151.6 ± 22	147.7 ± 20	0.42
**BSA (m** ^**2**^ **)**	**1.62 [0.27]**	**1.38 [0.38]**	0.039*
*Clinical parameters*			
Urea (mg/dL)	13.3 ± 5.4	11.6 ± 7.4	0.14
**Creatinine (mg/dL)**	**0.8** ± **0.2**	**0.6 ± 0.3**	0.011*
Total bilirubin (mg/dL)	0.9 ± 0.5	1.16 ± 0.5	0.14
Direct bilirubin (mg/dL)	0.2 ± 0.2	0.2 ± 0.2	0.72
SGOT (U/L)	40.3 ± 28.5	39.2 ± 16.6	0.84
SGPT (U/L)	36.7 ± 17.8	33.3 ± 12.6	0.34
ALP (U/L)	84	66.25	0.75

Data are presented as mean ± SD or median [IQR] for continuous variables and *n* (%) for categorical variables. BSA, body surface area; SGOT, serum glutamic oxaloacetic transaminase; SGPT, serum glutamic pyruvic transaminase; ALP, alkaline phosphatase.

*p ≤ 0.05. Bold values indicate statistically significant differences between groups.

### Intraoperative data

Both groups were similar in intraoperative characteristics (Table 2).

**Table 2:** Intra-operative characteristics of pulsatile and non-pulsatile CPB groups.

Intra-operative variables	Pulsatile group (*n* = 39)	Non-pulsatile group (*n* = 38)	*p*-Value
CPB time (min)	52 [42]	62.5 [51]	0.54
Aortic cross-clamp time (min)	31 [37]	47.5 [47]	0.44
Urine output (mL)	270 [300]	205 [293]	0.93
pH	7.38 [0.09]	7.39 [0.1]	0.38
PO_2_ (mm of Hg)	253 [138.3]	285 [132.4]	0.13
PCO_2_(mm of Hg)	40 [11.1]	36.7 [15]	0.34
HCO_3_(mmol/L)	22.3 ± 2.8	22.7 ± 2.2	0.45
Lactate	2 [1.7]	2 [0.75]	0.68

Data are presented as mean ± SD for normally distributed variables and median [IQR] for non-normally distributed variables. No *p*-values reached statistical significance (*p* ≤ 0.05). Abbreviations: CPB, cardiopulmonary bypass; PO_2_, partial pressure of oxygen; PCO_2_, partial pressure of carbon dioxide; IQR, interquartile range.

### Postoperative data

All postoperative characteristics were similar across both groups except for higher urea (*p*-value 0.049) in the PP group (median = 14, IQR = 8.3) compared to the NP group (median = 12, IQR = 4.3) (Table 3).

**Table 3:** Post-operative characteristics of pulsatile and non-pulsatile CPB groups.

	Pulsatile group (*n* = 39)	Non-pulsatile group (*n* = 38)	*p*-Value
*Vasopressor and inotrope*			
Adrenaline (hour)	0 [17]	0 [18]	0.64
Noradrenaline(hour)	4.5 [20]	7.5 [23]	0.65
Milrinone (hour)	0 [20]	0 [21]	0.93
Vasopressin (hour)	0 [0]	0 [0]	0.63
Phenylephrine (hour)	0 [0]	0 [0]	0.63
*Laboratory parameters*			
**Urea (mg/dL)**	**14 [8.3]**	**12 [4.3]**	0.049*
Creatinine (mg/dL)	0.86 [0.82]	0.8 [0.47]	0.30
Total bilirubin	1 [0.9]	0.7 [0.9]	0.08
Direct bilirubin	0.3 [0.4]	0.2 [0.3]	0.19
SGOT (U/L)	38 [19.3]	36.5 [27.3]	0.73
SGPT (U/L)	61 [36.5]	60.5 [24]	0.91
ALP (U/L)	75.5 [47.3]	84.5 [100.8]	0.73
Lactate 1st postop	3 [3.6]	4 [3]	0.21
Lactate 2nd postop	1.9 [2.2]	2.4 [2.2]	0.21
*Clinical parameters*			
Urine output 1st post-op (mL)	2680 [964]	2532 [1301]	0.91
Urine output 2nd post op (mL)	1952 [3270]	2041 [2839]	0.74
Drain output 1st day (mL)	300 [330]	230 [328]	0.57
Drain output 2nd day (mL)	0 [98]	20 [105]	0.43
ICU stay (days)	2 [2]	2 [2]	0.55
Ventilator support (hours)	4 [5]	4.5 [4]	0.74
Hospital stay (days)	7 [5]	8 [5]	0.14
*Complications*			
Heart failure	0	1 (2.63%)	0.49
Chest infection	0	1 (2.63%)	0.49
Re-exploration	2 (5.12%)	3 (7.89%)	0.98
Neurological complications	2 (5.12%)	0	0.49
Atrial fibrillation	0	2 (5.26%)	0.24
Surgical infection	2 (5.12%)	1 (2.63%)	1
Mortality	2 (5.12%)	2 (5.26%)	1

Data are shown as median [IQR] for continuous variables and *n* (%) for categorical variables unless otherwise specified. Mean ± SD is used only for normally distributed parameters. *p ≤ 0.05 indicates statistical significance (bolded). Abbreviations: SGOT, serum glutamic oxaloacetic transaminase; SGPT, serum glutamic pyruvic transaminase; ALP, alkaline phosphatase; IQR, interquartile range; CPB, cardiopulmonary bypass.

## Discussion

Our findings suggest that in low-risk patients undergoing shorter elective procedures, the clinical advantage of pulsatile perfusion is likely to be small. This study was not specifically powered to detect microcirculatory differences, and the absence of a measurable benefit may be related to the characteristics of our cohort and operative context. Most participants were young and had few comorbidities, and CPB and aortic cross-clamp times were relatively short, limiting ischemic stress and reducing the opportunity for pulsatile flow to translate into clinically evident gains. In this regard, our results are consistent with previous reports that did not demonstrate significant differences in major organ outcomes between pulsatile and non-pulsatile perfusion [13, 14]. At the same time, the overall literature may over-represent studies suggesting benefit, as trials with favorable or positive results are more likely to be published than those with neutral or negative findings, which could contribute to an under-recognition of the non-inferiority of non-pulsatile flow in lower-risk settings.

Several technical aspects of the pulsatile perfusion used in this study may also help to explain the absence of measurable benefit. Roller-pump–generated pulsatility produces a relatively simplified and non-physiologic waveform compared with dedicated systems, which may limit potential microcirculatory advantages [13]. In addition, the target pulse pressure of ≥15 mm Hg, although sufficient to maintain acceptable mean arterial pressure, may have been too low to effectively transmit pulsatility to the microvascular bed [6, 13, 14]. Shorter CPB duration and cross-clamp time may have further reduced the exposure to potentially beneficial shear-stress and flow characteristics. Prior experimental and clinical work has suggested that the magnitude, waveform quality, and duration of pulsatile support are key determinants of microvascular responsiveness and end-organ protection [6, 13–15].

Although baseline BSA and serum creatinine values were significantly higher in pulsatile than non-pulsatile perfusion, both parameters remained within normal physiological ranges. Due to the relatively small sample size and the absence of clinically relevant postoperative renal differences, adjusted multivariable analyses were not performed, as such models may be prone to overfitting and yield unstable estimates. This should be considered when interpreting the renal outcomes. Patients in the non-pulsatile CPB group had a nonsignificant increase in CPB time, findings similar to those from a previous study [16]. Lactate concentration is a good indicator of non-optimal tissue perfusion. Hyperlactatemia can occur in up to 20% of all CPB patients and is associated with an increased risk of morbidity and mortality [17, 18]. Likewise, the urine output was similar between the groups, aligning with findings of a previous study [19]. Lactate was lower in pulsatile groups, but the difference was not statistically significant. Chiu et al. reported that hepatic function is preserved with pulsatile blood flow during CPB, as reflected in postoperative SGOT levels [20]. However, in our study, postoperative LFT parameters were similar between the two groups.

The number of patients requiring inotropic or vasopressor support did not differ between the two groups during the postoperative period, consistent with previous findings [6]. Although blood urea levels were significantly higher in the pulsatile group than in the non-pulsatile group, creatinine and urine output were comparable, indicating preserved overall renal function. This may reflect factors beyond baseline renal function, such as preoperative renal perfusion, intraoperative hemodynamic variations, or differences in circuit flow characteristics that were not captured. Subclinical hemolysis is a potential contributor, as hemolysis can increase nitrogen load and urea production despite preserved creatinine and urine output. Prior studies have reported increased hemolysis and a higher risk of gaseous microembolism with pulsatile perfusion [21, 22, 23]. Contemporary work highlights that renal oxygenation and microcirculatory perfusion may respond differently to perfusion conditions during CPB, supporting the value of mechanistic endpoints and sensitive renal biomarkers in future trials [24]. Future studies should therefore incorporate direct assessments of renal perfusion together with structured hemolysis profiling, including plasma-free hemoglobin, lactate dehydrogenase, haptoglobin, and other sensitive renal and hemolysis-related biomarkers measured at predefined time points, to better delineate the mechanisms underlying these biochemical differences.

This study focused on broad clinical outcomes such as ICU stay, hospital stay, liver and renal function tests, and postoperative complications, which are relevant in a low-resource setting like ours. However, microcirculatory and organ-specific perfusion parameters were not assessed due to limited laboratory resources and financial constraints. These parameters often serve as mechanistic indicators of how pulsatile flow might influence organ-level physiology, and their omission constrains interpretation of the neutral findings. Future studies incorporating such microcirculatory or biochemical endpoints would help clarify whether pulsatile perfusion confers physiological benefits that are not reflected in routine postoperative laboratory values or length-of-stay metrics. The sample size calculation was based on case prevalence rather than effect size, which is a methodological limitation. Consequently, formal post-hoc power analysis for the primary outcomes was not performed, as achieved power estimates based on observed effect sizes may be misleading. This limitation should be considered when interpreting the absence of statistically significant differences between groups, and larger, multicenter studies with predefined effect-size–based calculations are needed to confirm these results. The vasoactive-inotropic score was not calculated perioperatively or postoperatively. Instead, we recorded the duration and type of vasopressor and inotropic support administered.

In low-resource cardiac centers, these findings suggest that routine adoption of pulsatile CPB may not be necessary for low-risk elective procedures when standard non-pulsatile perfusion is safely and consistently implemented.

## Conclusion

Our study suggests that pulsatile CPB does not confer significant advantages over non-pulsatile CPB in perioperative outcomes in elective cardiac surgeries in younger patients without significant comorbidities. Larger, well-designed randomized controlled trials focusing on high-risk patient populations are warranted to validate these observations and determine the clinical relevance of pulsatile flow in cardiac surgery.

## Data Availability

Original data is available from the corresponding author on reasonable request.
